# Six Weeks of Boxing Training Lowers Blood Pressure and Improves Vascular Function in Young Men and Women with Elevated Blood Pressure or Stage 1 Hypertension †

**DOI:** 10.3390/sports14010005

**Published:** 2026-01-01

**Authors:** Francisco Morales-Acuna, Manuel Gomez, Matías Monsalves-Álvarez, Lisa Rodriguez, Paulina Caraveo, Alvaro N. Gurovich

**Affiliations:** 1 Especialidad en Medicina del Deporte y La Actividad Física, Facultad de Ciencias Médicas, Universidad de Santiago de Chile, Estación Central, Santiago 9170022, Chile; 2 Clinical Applied Physiology (CAPh) Laboratory, Department of Physical Therapy and Movement Sciences, College of Health Sciences, The University of Texas at El Paso, El Paso, TX 79968, USAagurovich@utep.edu (A.N.G.); 3 Clinica MEDS, Lo Barnechea, Santiago 7691236, Chile; 4 Exercise and Rehabilitation Science Institute, Faculty of Rehabilitation Sciences, Universidad Andrés Bello, Las Condes, Santiago 7591538, Chile; matias.monsalves@unab.cl; 5 Geroscience Center for Brain Health and Metabolism (GERO), Ñuñoa, Santiago 7800003, Chile

**Keywords:** boxing training, high blood pressure, endothelial function

## Abstract

(1) Background: Early stages of hypertension, including elevated blood pressure and stage 1 hypertension, are known to increase cardiovascular risk and mortality. Exercise is widely recommended for blood pressure management; however, the optimal exercise modality and the underlying vascular mechanisms remain uncertain. (2) Methods: This study investigated the effects of boxing training on clinical and vascular parameters in young adults with elevated blood pressure or stage 1 hypertension. Twenty-four participants (mean age 25.4 ± 4.9 years) were randomly assigned to a boxing training group or a control group. Over six weeks, the intervention group performed boxing training three times per week, consisting of ten three-minute rounds interspersed with one-minute rests, while controls completed flexibility and balance exercises. (3) Results: Boxing training produced significant group-by-time interactions for peripheral and central blood pressure, endothelial function, and carotid artery structure. Reductions were observed in systolic (~16 mmHg) and diastolic (~10 mmHg) blood pressure and in central systolic pressure (~mmHg). Vascular function improved, as indicated by greater brachial and popliteal flow-mediated dilation and increased forearm and calf blood flow, accompanied by enhanced nitric oxide bioavailability and reduced inflammation. (4) Conclusions: These findings suggest that boxing training is an effective and time-efficient exercise modality for improving vascular health and managing early hypertension in young adults.

## 1. Introduction

Cardiovascular diseases (CVD), such as coronary artery disease, heart failure, peripheral artery disease, and stroke, are responsible for 17.3 million annual deaths, which account for a third of all global causes of death [1]. High blood pressure is the most prevalent risk factor for the development of CVD and represents the primary cause of global morbidity and mortality [2]. Even early stages of high blood pressure, such as elevated blood pressure or stage 1 hypertension, have been shown to increase cardiovascular mortality [3].

While physical inactivity is a significant contributor, the etiology of hypertension is complex and multifactorial, involving lifestyle factors such as poor diet, excessive body mass index (BMI), high visceral adiposity, and the chronic effects of stress or poor sleep hygiene. Several pathological features are involved and interconnected in the development of high blood pressure, such as endothelial dysfunction, oxidative stress, inflammation, autonomic dysfunction, an overreactive renin–angiotensin–aldosterone pathway, and arterial stiffness [4]. Endothelial dysfunction appears to have a central role in the progression to high blood pressure according to the Mosaic Theory, mainly by affecting the ability of the vessels to maintain homeostasis [5]. Endothelial dysfunction can be identified by one or more of the following characteristics: (1) a decline in nitric oxide (NO) bioavailability and subsequent impaired vasodilation, (2) upregulation of adhesion molecules and inflammatory genes, (3) oxidant stress exacerbation, and (4) increased permeability of the endothelial barrier [6]. Meanwhile, arterial stiffness directly impacts blood pressure by affecting afterload and arterial–ventricular coupling [7,8].

Individuals with elevated blood pressure or stage 1 hypertension without an estimated 10-year risk of CVD of ≥10%, as calculated by the American College of Cardiology/American Heart Association (ACC/AHA) Pooled Cohort Equations, are not candidates for drug treatment. In turn, management strategies for this population primarily include lifestyle modifications, such as the incorporation of an exercise training program [9]. Current medical guidelines have established that exercise is a cornerstone in high blood pressure prevention and treatment, and its effectiveness is comparable to drug treatment [9,10]. Indeed, for every dollar invested in strategies to incorporate physical activity as a preventive cardiovascular tool, there is a return of approximately three to six dollars in terms of healthcare costs [1]. However, the optimal exercise prescription dose and exercise modality for high blood pressure and the mechanisms behind the health benefits of exercise have not yet been fully clarified [11].

Boxing training is a type of exercise that involves high cardiovascular demands, high-impact punching, and coordinated movements in an enjoyable environment. It is characterized by alternating periods of intense physical effort (e.g., striking drills, footwork) and brief, active recovery periods. This interval nature demands significant and rapid engagement of both the circulatory and respiratory systems, which may be a potent stimulus for inducing central and peripheral cardiovascular adaptations [12,13]. Previously, boxing training has been shown to have excellent motor outcomes in stroke [14] and Parkinson’s disease patients [15]. Nonetheless, research regarding the effects of boxing training on cardiovascular health is scarce. To date, no randomized controlled trial employing boxing training as an intervention for individuals with high blood pressure has been conducted. Therefore, this study aims to determine the effects of boxing training on clinical and vascular health in individuals with elevated blood pressure or stage 1 hypertension. We hypothesized that six weeks of high-intensity boxing training would lead to significant reductions in both peripheral and central blood pressure, alongside improvements in endothelial function, arterial stiffness, and inflammation, compared to a non-exercise control group.

## 2. Materials and Methods

### 2.1. Participants

Participants were recruited from the University of Texas at El Paso and its surroundings. A preliminary blood pressure screening and a health questionnaire identified them. The cohort consisted of subjects who met the following inclusion criteria: (1) ≥18 years old, (2) systolic blood pressure (SBP) between 120 and 139 mmHg and/or diastolic blood pressure (DBP) between 80 and 89 mmHg obtained from 2 different days, (3) an estimated 10-year risk of CVD ≤ 10%, calculated by the ACC/AHA Pooled Cohort Equations, and (4) no current participation in 3 or more days per week of endurance or resistance exercise training. Exclusion criteria included non-controlled cardiac, pulmonary, or metabolic diseases, smoking, consumption of nutritional supplements containing antioxidants, and any physical impairment to exercise. All participants signed a written informed consent. The study protocol was approved by the local ethics committee (UTEP IRB Study Number 1364179-3).

### 2.2. Protocol

The study was a double-blind (both evaluator and participant), randomized, controlled trial (ClinicalTrials.gov ID NCT06413251). An initial brachial blood pressure screening was performed to identify potential participants for the present study. Those with SBP readings between 120 and 139 mmHg or DBP between 80 and 89 mmHg were instructed to report to the Clinical Applied Physiology Lab at the University of Texas at El Paso for a second blood pressure assessment to confirm the diagnosis of elevated blood pressure or stage 1 hypertension. Then, participants were asked to complete a health questionnaire to rule out any cardiac, pulmonary, or metabolic conditions. Once they were cleared to participate, participants started the informed consent process. After obtaining the consent, their height and weight were measured using a stadiometer (Seca 225, Seca Medical Measuring Systems and Scales, Hamburg, Germany) and a digital scale (Tanita WB-110A, Tanita Corporation, Tokyo, Japan). Thereafter, participants were asked to lie down in supine position over an examination table for 10 min, where carotid–femoral tonometry (Sphygmocor, Xcel, West Ryde, Australia), carotid, brachial, and popliteal ultrasound imaging (MyLab30 Gold Cardiovascular, Esaote, Firenze, Italy), and strain gauge venous occlusion plethysmography (AI6 Arterial Inflow System, D.E. Hokanson Inc., Bellevue, WA, USA) were performed from the right side of their body. At the end of the lab visit, blood samples were collected from the antecubital vein of the participants. All vascular measurements were conducted under strictly standardized conditions in a climate-controlled room (22–24 °C). Participants reported to the laboratory in the morning (7:00 a.m.–12:00 p.m.) after a 12 h overnight fast and were instructed to abstain from alcohol, caffeine, and vigorous exercise for at least 24 h prior to the assessment.

Participants were then randomly assigned by a member of the research team (L.R.), who was not involved in the assessments or the analysis, into a boxing or a control group using an online number generator (https://www.graphpad.com/quickcalcs/randomize1/ accessed on 20 August 2019). Participants were unaware of the allocation possibilities. A 6-week intervention was designed for each group at the Clinical Applied Physiology Lab at the University of Texas at El Paso. Each intervention was supervised by members of the research team who were not involved in the assessments or the analysis (M.G. and P.C.). Finally, all the measurements taken in the first lab visit were repeated in the same order at the end of the intervention. The evaluator (F.M.A.) who performed the pre- and post-assessments was blinded to the allocation of each participant.

### 2.3. Measurements

#### 2.3.1. Brachial Blood Pressure

Blood pressure assessments were performed according to the ACC/AHA recommendations [9]. Briefly, participants were seated in a chair for five minutes with their feet on the floor and their back supported, followed by an attended recording of blood pressure in triplicate with a validated automated brachial blood pressure cuff (BP760, Omron Healthcare, Inc., Lake Forest, IL, USA).

#### 2.3.2. Central Blood Pressure

A cuff-based device for PWA (Sphygmocor Xcel, AtCor Medical, West Ryde, Australia) was attached to the right arm of every participant in a supine position after 10 min of resting. Each PWA assessment lasted between 60 and 120 s: 50–110 s to record brachial blood pressure and 10 s of sub-diastolic recordings. Central blood pressure waveforms were generated by a validated transfer function [16]. According to the manufacturer’s recommendations, the blood pressure waveforms were considered acceptable if the overall quality control indices were equal to or above 75%. The following PWA variables were assessed: central systolic blood pressure (cSBP), central diastolic blood pressure (cDBP), central pulse pressure (cPP), augmentation index (AIx), and augmentation index normalized to a heart rate of 75 bpm (AIx@75). cSBP and cDBP are the estimated blood pressures at the ascending aortic wall during systole and diastole, respectively. cPP is the difference between cSBP and cDBP. AIx is the ratio between the increase in aortic blood pressure produced by reflected waves in relation to cPP, and AIX@75 is the normalization of AIx to 75 bpm [17]. All these variables have been associated with cardiovascular risk [18]. Additionally, Wasted Left Ventricular Energy (LVE_W_), a surrogate for left ventricle work and myocardial oxygen demand, was estimated from the pulse pressure curve and the duration of the reflected wave. LVE_W_ was calculated using the following formula: LVE_W_ = ((ED − Δt_p_) × (cSBP − Pi) π/4) × 1.33322, where ED is ejection duration, Δt_p_ is time to arrival of the reflected pressure wave, and Pi is the pressure at the first inflection point marking the onset of the reflected aortic pressure wave [19,20].

#### 2.3.3. Pulse Wave Velocity

Carotid–femoral pulse wave velocity (PWVcf) is considered the gold standard to determine arterial stiffness [21]. For this procedure, participants were supine over an examination table for 10 min, and a cuff was placed on the right thigh. During this period, carotid and femoral pulses were palpated. Then the following distances were measured using a metallic tape: carotid to sternal notch, sternal notch to cuff, and femoral to cuff. Once the participant reached hemodynamic stability, a high-fidelity tonometer was placed over the carotid artery. Pulse waves of the carotid and femoral arteries were simultaneously recorded by the tonometer and the cuff, respectively. PWVcf was determined by dividing the distance obtained through the subtractive method by the delay time between waves [22,23,24,25].

#### 2.3.4. Flow-Mediated Dilation

Flow-mediated dilation (FMD) is considered a surrogate of endothelial function. For brachial FMD, participants were asked to lie down in a supine position over an examination table for at least 10 min. A total of six electrodes were attached to their chest in the standard lead II setting, where three electrodes were connected to a high-definition ultrasound machine (MyLab30 Gold Cardiovascular, Esaote, Firenze, Italy) and the other three were connected to an electrocardiogram trigger system (MP150WSW, BIOPAC Systems Inc., Goleta, CA, USA and Frame Grabbing and Digital Data Input modules, Medical Imaging Applications LLC, Coralville, IA, USA). The right arm was moved at 80–90° of shoulder abduction over an armrest, and a blood pressure cuff was attached to the forearm just below the antecubital fossa. A 12 MHz linear phase array ultrasound transducer (LA435, Esaote, Firenze, Italy) with a transducer holder (Patent US20200155117A1) was placed 5 cm above the antecubital fossa to image the right brachial artery according to international guidelines for FMD [26,27,28]. Basal artery diameters and peak systolic blood flow velocities were recorded in basal conditions at every QRS complex captured by the electrocardiogram trigger system using an automated edge-detection software (Vascular Research Tools, version 5, Medical Imaging Applications LLC, IA, USA) for 30 s. Then, the forearm cuff was inflated to supra-systolic pressure (>200 mmHg) for 5 min, followed by the deflation of the cuff. Brachial artery diameters and blood flow velocities were continuously registered every 3 s for 150 s, starting at 30 s before deflation until 2 min after deflation using the automated edge-detection software. Peak diameters were identified as the single peak diameter observed during the plateau phase after cuff deflation [29]. The reliability of FMD analysis in our lab has already been tested and is described elsewhere [30]. For popliteal FMD, the same procedure is repeated, but with the difference that the cuff and the transducer are placed in the leg and in the popliteal fossa, respectively. FMD was calculated as FMD% = 100 × (peak diameter − basal diameter)/basal diameter [31].

#### 2.3.5. Forearm and Calf Blood Flow

Forearm blood flow (FBF) and calf blood flow (CBF) were assessed by strain gauge venous occlusion plethysmography (AI6 Arterial Inflow System, D.E. Hokanson Inc., Bellevue, WA, USA). Participants were in a supine position over an examination table where strain gauges were placed at the widest part of the right forearm or calf. Next, an upper-arm cuff for FBF and a thigh cuff for CBF were cycled from 0 to 50 mmHg for seven seconds every 15 s to prevent venous outflow. One minute before each measurement, a wrist and an ankle cuff were inflated to 200 mmHg to occlude hand and ankle circulation, respectively. Absolute blood flow was determined by the rate of change in limb circumference (e.g., slope) during the seven-second venous occlusion. FBF and CBF were estimated as the average of three readings in a minute [32].

#### 2.3.6. Forearm and Calf Blood Flow During Reactive Hyperemia

FBF and CBF during reactive hyperemia were measured after five minutes of occlusion of the arm or thigh, respectively. These measurements are a reliable non-invasive alternative to estimate endothelial function in resistance vessels [33]. Baseline FBF and CBF were recorded for 2 min, then the respective cuff was inflated to 200 mmHg for five minutes and then rapidly deflated. FBF and CBF were measured every 15 s for three minutes. Peak FBF and CBF were selected from the highest value of blood flow following deflation of the cuff [32].

#### 2.3.7. Carotid Artery Ultrasound

An 8–18 Hz ultrasound transducer (LA435, Esaote, Firenze, Italy) inserted into a neck transducer holder was attached to the right side of the neck of each participant, and the mid-section of the common coronary artery was identified in a high-definition ultrasound machine (MyLab30 Gold Cardiovascular, Esaote, Firenze, Italy). Resting arterial diameters and peak systolic blood flow velocities were recorded continuously for 10 s at a rate of 10 frames per second using an automated edge-detection software (Vascular Research Tools, Version 5, Medical Imaging Applications LLC, Coralville, IA, USA).

#### 2.3.8. Endothelial Shear Stress Estimations

Resting endothelial shear stress (ESS) of each artery was estimated during 10 seconds by Womersley’s approximation, using ESS = μ × SR and SR = 2K × V/D, where μ is blood viscosity, SR is shear rate, V is peak systolic velocity, D is artery diameter, K is a complex factor dependent only on the Womersley parameter (α), and α = (D/2) × (ω/(μ/ρ))^1/2^, where ω is the angular frequency of the flow pulsation (ω = freq × 2π), ρ is blood density, and μ is blood viscosity [34,35]. ESS is expressed in dynes/cm^2^. Blood viscosity and density were calculated using the following formulas [36,37,38]: $\mu_{plasma}=\frac{\exp\left[-5.64+\frac{1800}{T+273}\right]}{SR}$, $\mu =\mu_{plasma}\times exp(2.31HCT)$, and $\rho =[1.09HCT+1.035\times \left(1-HCT\right)]$, where μ_plasma_ is plasma dynamic viscosity expressed in Pa·s, T is temperature expressed in °C, and Hct is Hematocrit expressed as a fraction.

#### 2.3.9. Antecubital Vein Blood Collection

After 48 h of a low-nitrate diet under fasting conditions, 20 mL blood samples were obtained from the antecubital vein of each participant. Blood samples were immediately centrifuged to obtain plasma samples, with the latter being placed in aliquots at −80 °C until analysis. NOx (Total Nitric Oxide and Nitrate/Nitrite Parameter Assay Kit, R&D Systems, Minneapolis, MN, USA), lipid profile (HDL and LDL/VLDL Cholesterol Assay Kit, ab65390, Abcam, Cambridge, UK), CRP (C-Reactive Protein (human) ELISA Kit, 10011236, Cayman, Ann Arbor, MI, USA), IL-6 (Interleukin-6 (human) ELISA Kit, 501030, Cayman), TNF-α (TNF-α (human) ELISA Kit, 589201, Cayman), F2-isoprostanes (STAT-8-isoprostane ELISA Kit, 500431, Cayman), TAC (Antioxidant Assay Kit, 709001, Cayman), and SOD (Superoxide Dismutase Assay Kit, 706002, Cayman) were performed according to the manufacturer’s instructions for their respective kit at the core lab of the health sciences building at UTEP.

### 2.4. Interventions

#### 2.4.1. Boxing Training

First, during a familiarization visit, participants learned how to wrap their hands and perform basic boxing techniques, such as stance and punches, while wearing 14-oz gloves. This visit finished with an incremental boxing test that consisted of punching a 100 lb heavy bag (Ringside soft-filled leather, Ringside-CSI Fitness 1st, Lenexa, KS, USA) at a fixed force (~20 kg) and with an increase in the punching cadence every 2 min. The force was tracked by a sensor attached to the bottom of the heavy bag (UFC Force Tracker), which, via a phone app (XFORCE tracker, SEROSE), gave visual feedback of power and rhythm to the participant. The test started at a cadence of 140 punches per minute (ppm) controlled by a metronome (Pro Metronome by EUMLab, Xanin Tech. GmbH., Berlin, Germany) and was increased by 30 ppm every 2 min until fatigue. Fatigue was determined when participants were unable to maintain the punching tempo, and they were asked to perform a 1 min all-out effort to finish the test. Oxygen uptake (VO_2_) was measured during a subsample of sessions via indirect calorimetry using a portable metabolic cart (Parvomedics Inc., Sandy, UT, USA) at the end of each 2 min workload. Heart Rate (HR) was monitored continuously with a chest strap monitor (Polar H9). Rate of Perceived Exertion (RPE) was assessed immediately following each round using the 0–10 Modified Borg Scale.

The boxing training intervention consisted of three exercise sessions per week on nonconsecutive days for six weeks. All boxing training sessions were conducted at the Clinical Applied Physiology Lab, University of Texas at El Paso. Training sessions were individualized and were instructed by at least 2 lab members with a bachelor’s degree in kinesiology and experience in boxing training to ensure safety, proper technique, and adherence to the high-intensity interval protocol. The workout began with a 3 min warm-up period where participants actively moved their shoulders, elbows, wrists, and finger joints. Participants were then instructed to complete 10 rounds of three minutes with a one-minute resting period interspersed. During the rounds, participants punched a heavy bag (e.g., straight, jab, hook) or did mitt work. The intensity of the first 3 rounds was set at 90–95% VO_2_max and at approximately 95% heart rate reserve (HRR), which was above their individual ventilatory threshold 2. The intensity of the remaining seven rounds was set just below their first ventilatory threshold at approximately 60% of HRR or less than 4 on the modified RPE scale (1–10). The exercise intensity was deliberately tapered after the initial three high-intensity rounds (targeting 95% of HRR) to incorporate an active recovery and to manage accumulated fatigue. This varied intensity structure was implemented to improve exercise adherence over the six-week program and reduce the risk of overtraining. The final rounds were maintained at a moderate-intensity level (targeting 60% HRR), which is sufficient to sustain metabolic activity while promoting recovery. Heart rate and RPE were constantly monitored to ensure that each participant was exercising at the desired intensity. Training progression was adjusted weekly by increasing the punching cadence to maintain the target 95% HRR for the first three rounds and an RPE just below 4 for the remaining rounds.

#### 2.4.2. Flexibility and Balance Training (Control)

The control group performed 10 min of dynamic articular movement, 5 min of unipedal stance, and 5 min of stretching of the upper limbs three days per week for six weeks.

### 2.5. Statistical Analysis

Data was analyzed using SPSS version 24.0. Graphs were elaborated using GraphPad Prism 10.3.1. Normal distribution of the data was examined with the Shapiro–Wilk test and visual inspection. Independent *t*-tests were conducted to compare demographic variables between groups. A repeated measure general linear model (GLM) with two levels of time (pre and post) and using two groups (Boxing and Control) was employed to compare between-group and within-group differences. Fisher’s Least Significant Difference (LSD) was selected as the post hoc test and partial eta-squared effect size ($\eta_{p}^{2}$) as the indicator of effect size. $\eta_{p}^{2}$ of 0.02, 0.13, and 0.26 were considered small, medium, and large effects, respectively [39]. To evaluate whether sex or adiposity could confound the effects of the intervention, we conducted correlation analyses examining the associations between sex, baseline body mass index (BMI), changes in BMI (ΔBMI), and hemodynamic, vascular, and biochemical responses. Baseline BMI was calculated using measured height and pre-intervention body mass. Pearson correlation coefficients (r) and corresponding *p*-values were computed for each predictor–outcome pair. Significance was established at α ≤ 0.05. The sample size was determined using the software G*Power 3.1. Based on Izadi et al.’s [40] results on the effects of six weeks of HIIT on SBP (effect size = 1.73), establishing α at 0.05 and β at 0.2, and assuming a 30% dropout rate, the estimated number of participants per group was 12.

## 3. Results

**Figure 1:**
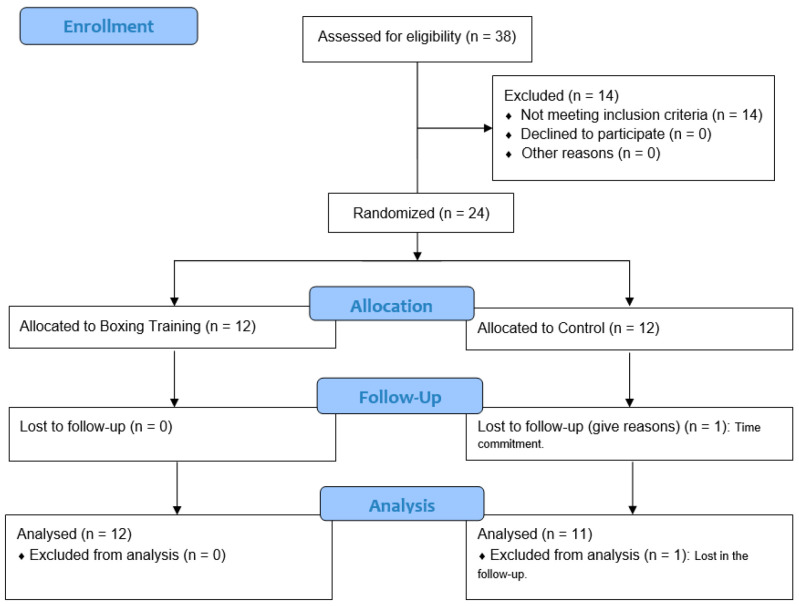
Flow chart of participants.

Initially, 38 individuals were assessed for eligibility in a time frame of approximately 3 months. Fourteen subjects were excluded because their blood pressure did not meet the inclusion criteria. As Figure 1 exhibits, a total of 24 participants were randomly allocated to one of the two groups. Only 1 participant was lost to follow-up assessments in the control group, leaving 12 participants in the boxing group and 11 participants in the control group that were finally analyzed. All analyzed participants were college students.

All variables were normally distributed except for age. All data is presented as mean and standard deviation, unless otherwise stated. The baseline demographic characteristics of both groups are summarized in Table 1. No difference was found between groups regarding age (U = 86.0, *p* = 0.235), height (t(21) = 0.39, *p* = 0.698), weight (t(21) = 0.78, *p* = 0.442), hematocrit (t(21) = −0.06, *p* = 0.953), SBP (t(21) = −0.98, *p* = 0.339), and DBP (t(21) = −0.301, *p* = 0.766) before the intervention.

Compliance with the boxing training protocol was 98.1% ± 3.9%. Overall, 95.4% of the target heart rates were sustained during the three high-intensity rounds. Furthermore, they kept their heart rate within the required range for low intensity or recovery in all (100%) of the measurements taken during the remaining 7 rounds. Additionally, participants reported a mean RPE of 8.3 and 2.8 for the high-intensity and low-intensity rounds, respectively. Meanwhile, the compliance with the flexibility and balance training was 27.3% ± 13.4% in the control group. No adverse cardiovascular effects were reported throughout the intervention. Only 3 participants experienced anterior shoulder tenderness when punching during the first week of training, which was relieved in the subsequent weeks.

### 3.1. Peripheral Blood Pressure

**Figure 2:**
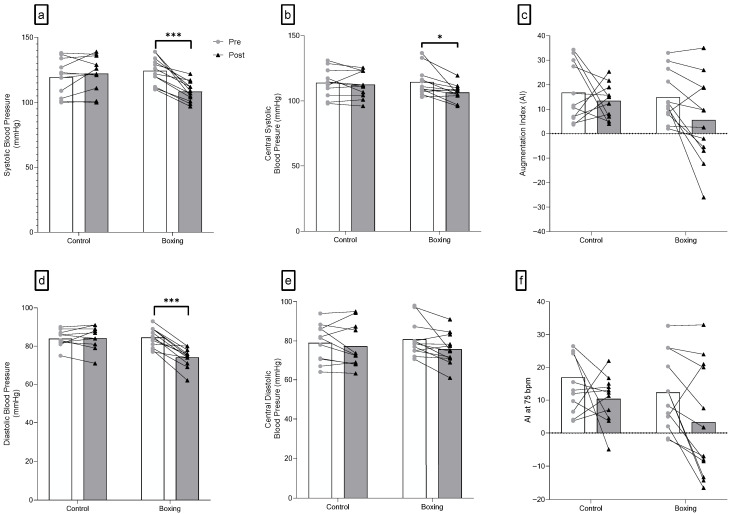
Peripheral (**a**,**d**) and central blood pressure (**b**,**c**,**e**,**f**) changes following 6 weeks of boxing training in individuals with elevated blood pressure or stage 1 hypertension. Augmentation Index (AI) represents the central aortic pressure waveform, showcasing hoy a reflected pulse wave adds to the forward wave. *** *p* < 0.001; * *p* < 0.05.

Significant interactions were observed for SBP (F(1,21) = 32.34, *p* < 0.001) and DBP (F(1,21) = 39.54, *p* < 0.001). No differences were observed in SBP (F(1,21) = 0.96, *p* = 0.339) and DBP (F(1,21) = 0.09, *p* = 0.766) between groups at baseline. Six weeks of boxing training largely decreased systolic blood pressure (F(1,21) = 48.89, *p* < 0.001, $\eta_{p}^{2}$ = 0.700) and diastolic blood pressure (F(1,21) = 78.48, *p* < 0.001, $\eta_{p}^{2}$ = 0.789) in individuals with elevated blood pressure and stage 1 hypertension (Figure 2 and Table 2).

### 3.2. Pulse Wave Analysis

Significant group-by-time interactions were only observed for cSBP (F(1,21) = 4.29, *p* = 0.05). Meanwhile, there were no significant interactions for cDBP (F(1,21) = 1.52, *p* = 0.231), cPP (F(1,21) = 0.52, *p* = 0.479), AIx (F(1,21) = 1.06, *p* = 0.314), AIx@75 (F(1,21) = 0.20, *p* = 0.658), and LVE_w_ (F(1,21) = 0.46, *p* = 0.504). A large reduction in cSBP (F(1,21) = 13.15, *p* = 0.002, $\eta_{p}^{2}$ = 0.385) was observed in the boxing training group following the intervention. Additionally, no significant group-by-time interaction was detected in the duration of the reflected wave (F(1,21) = 2.99, *p* = 0.098) (Figure 2 and Table 2).

### 3.3. Arterial Stiffness

There was no significant group-by-time interaction for PWVcf (F(1,21) = 0.30, *p* = 0.096) (Table 2).

### 3.4. Vascular Adaptations

**Figure 3:**
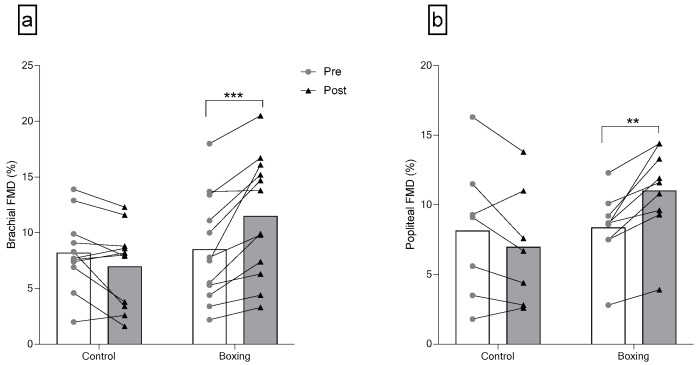
Brachial (**a**) and Popliteal (**b**) Endothelial Function after 6 weeks of boxing training in individuals with elevated blood pressure or stage 1 hypertension. FMD: flow-mediated dilation. *** *p* < 0.001; ** *p* < 0.01.

There was a significant group-by-time interaction for brachial FMD (F(1,21) = 22.46, *p* < 0.001). No differences were observed in brachial artery diameter (F(1,21) = 0.33, *p* = 0.573), resting brachial ESS (F(1,21) < 0.01, *p* = 0.967), and brachial FMD (F(1,21) = 0.03, *p* = 0.858) between groups at baseline. Six weeks of boxing training largely increased brachial FMD (F(1,21) = 23.58, *p* < 0.001, $\eta_{p}^{2}$ = 0.529) in individuals with elevated blood pressure or stage 1 hypertension. There were no significant group-by-time interactions for brachial artery diameter (F(1,21) = 3.05, *p* = 0.095) and resting brachial ESS (F(1,21) = 0.75, *p* = 0.398) (Figure 3 and Table 3).

Significant group-by-time interactions were observed for popliteal artery diameter (F(1,14) = 7.74, *p* = 0.015) and popliteal FMD (F(1,14) = 18.39, *p* = 0.001). No differences were observed in popliteal artery diameter (F(1,14) = 3.90, *p* = 0.068), resting popliteal ESS (F(1,14) = 1.35, *p* = 0.264), and popliteal FMD (F(1,14) = 0.03, *p* = 0.858) between groups at baseline. Six weeks of boxing training induced a large increase in popliteal artery FMD (F(1,14) = 20.19, *p* = 0.001, $\eta_{p}^{2}$ = 0.591) and popliteal artery diameter (F(1,14) = 7.53, *p* = 0.016, $\eta_{p}^{2}$ = 0.350). There was no group-by-time interaction for resting popliteal ESS (F(1,14) = 0.92, *p* = 0.354) (Figure 3 and Table 3).

There was a significant group-by-time interaction for carotid artery diameter (F(1,21) = 13.44, *p* = 0.001). No differences were observed in carotid artery diameter (F(1,21) = 3.43, *p* = 0.078) and resting carotid ESS (F(1,21) = 0.10, *p* = 0.750) between groups at baseline. A significant increase in carotid artery diameter (F(1,21) = 11.68, *p* = 0.003, $\eta_{p}^{2}$ = 0.357) was observed in the boxing group at the end of the intervention. There was no group-by-time interaction for resting carotid ESS (F(1,21) = 1.55, *p* = 0.228) (Table 3).

**Figure 4:**
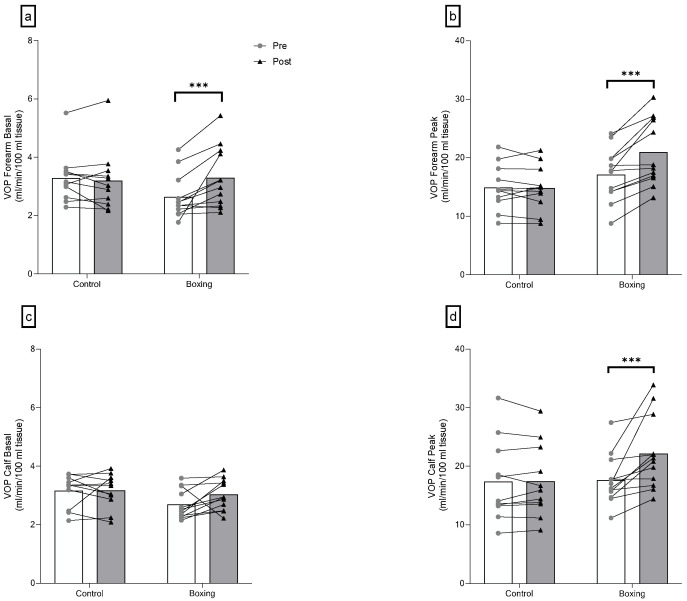
Plethysmography changes of the forearm (**a**,**b**) and the calf (**c**,**d**) following 6 weeks of boxing training in individuals with elevated blood pressure or stage 1 hypertension. VOP: venous occlusion plethysmography. *** *p* < 0.001.

There were significant group-by-time interactions for basal forearm blood flow (F(1,21) = 10.88, *p* = 0.003), peak forearm blood flow (F(1,21) = 20.17, *p* < 0.001), and peak calf blood flow (F(1,21) = 9.71, *p* = 0.005). No differences were observed in basal forearm blood flow (F(1,21) = 3.70, *p* = 0.068), peak forearm blood flow (F(1,21) = 1.53, *p* = 0.230), and peak calf blood flow (F(1,21) = 0.01, *p* = 0.912), while differences were found in basal calf blood flow (F(1,21) = 4.58, *p* = 0.044) between groups at baseline. Six weeks of boxing training led to a large increase in basal forearm blood flow (F(1,21) = 17.79, *p* < 0.001, $\eta_{p}^{2}$ = 0.459), peak forearm blood flow (F(1,21) = 39.94, *p* < 0.001, $\eta_{p}^{2}$ = 0.655), and peak calf blood flow (F(1,21) = 20.58, *p* < 0.001, $\eta_{p}^{2}$ = 0.495) (Figure 4 and Table 3).

### 3.5. Nitric Oxide Bioavailability

A significant group-by-time interaction was observed for NOx (F(1,20) = 10.08, *p* = 0.005). NOx was similar between groups at baseline (F(1,20) = 0.67, *p* = 0.424). A significant increase in NOx (F(1,20) = 8.37, *p* = 0.009, $\eta_{p}^{2}$ = 0.295) was found in the boxing group at the end of the intervention (Table 4).

### 3.6. Inflammation

A significant group-by-time interaction was observed for CRP (F(1,20) = 8.03, *p* = 0.01). No differences were observed in CRP (F(1,20) = 0.11, *p* = 0.745), IL-6 (F(1,20) = 0.30, *p* = 0.593), and TNF-α (F(1,18) = 0.23, *p* = 0.641) between groups at baseline. Six weeks of boxing training largely reduced CRP (F(1,20) = 13.53, *p* = 0.001, $\eta_{p}^{2}$ = 0.404). There were no significant group-by-time interactions for IL-6 (F(1,20) = 0.36, *p* = 0.851) and TNF-α (F(1,20) = 1.74, *p* = 0.203) (Table 4).

### 3.7. Oxidative Stress

There were no group-by-time interactions for 8-isoprostane (F(1,20) = 0.86, *p* = 0.364), SOD (F(1,20) = 0.12, *p* = 0.731), and TAC (F(1,20) = 2.23, *p* = 0.151). No differences were observed in 8-isoprostane (F(1,20) = 0.21, *p* = 0.649), SOD (F(1,20) = 0.07, *p* = 0.791), and TAC (F(1,20) = 0.16, *p* = 0.693) between groups at baseline (Table 4).

To examine whether sex or adiposity could influence the observed intervention effects, correlation analyses were conducted between sex, baseline BMI, ΔBMI, and primary outcomes (SBP Delta, DBP Delta, ΔB_FMD%, ΔP_FMD%, ΔNOx, and ΔCRP). The complete results are presented in Supplementary Table S1. Across all predictors, correlations were weak and did not reach statistical significance. Baseline BMI and ΔBMI showed small and non-significant associations with blood pressure, vascular function, and biochemical markers. Similarly, sex demonstrated only minor correlations with changes in FMD%, NOx, and blood pressure. These findings indicate that neither sex nor adiposity meaningfully influenced the primary outcomes and support the robustness of the intervention effects after adjusting for these covariates.

## 4. Discussion

The present study is the first randomized controlled trial to evaluate the effects of boxing training on brachial blood pressure, central blood pressure, arterial stiffness, vascular adaptations, nitric oxide bioavailability, inflammation, and oxidative stress in individuals with elevated blood pressure or stage 1 hypertension. The primary findings we discovered indicate that 6 weeks of boxing training among this population lead to improvements in (1) clinical outcomes, including peripheral and central blood pressure, and (2) vascular outcomes, specifically enhancing conduit artery endothelial function, resistance vessel structure and endothelial function, as well as carotid artery structure. These changes are associated with an increase in NO bioavailability and a decrease in inflammation.

### 4.1. Peripheral and Central Blood Pressure

The present study demonstrated that 6 weeks of boxing training induced a significant reduction in brachial SBP (~16 mmHg) and DBP (~10 mmHg) in individuals with elevated blood pressure or stage 1 hypertension. Similarly, Cheema et al. [12] reported 14 and 7 mmHg reductions in SBP and DBP, respectively, after 16 weeks of boxing training in adults with abdominal obesity. The findings of the present study are also in agreement with previous meta-analyses studying the effects of exercise on blood pressure. For example, Cornelissen et al. [10] reported an overall reduction in SBP/DBP of 3.5/2.5, 1.8/3.2, and 10.9/6.2 mmHg in endurance, resistance, and isometric training, respectively, in healthy individuals or those with high blood pressure. In addition, de Sousa et al. [41] reported 8.3 and 4.1 mmHg reductions in SBP and DBP, respectively, after resistance training in individuals with high blood pressure.

Furthermore, Inder et al. [42] reported 5.2 and 3.9 mmHg reductions in SBP and DBP, respectively, after isometric exercise training in healthy individuals or with high blood pressure. Moreover, Williamson et al. [43] reported 4.4 and 4.2 mmHg reductions in SBP and DBP, respectively, after a physical activity intervention in young adults with elevated blood pressure, stage 1 hypertension, or stage 2 hypertension. The SBP and DBP reductions described in those meta-analyses were lower than the reductions from the present study and the Cheema et al. study. The differences may rely on the intrinsic nature of boxing training, which is a whole-body physical activity but with an upper-body emphasis, that could induce more prominent local changes in the brachial artery (e.g., higher ESS). The blood pressure reductions following boxing training may be explained, in part, by an improvement in endothelial function, NO bioavailability, and a reduction in vascular inflammation and peripheral vascular resistance.

Lewington et al. [44] reported that a 10 mmHg reduction in SBP leads to a 30% decreased risk of coronary artery disease mortality and a 40% decreased risk of stroke mortality. In the same study, they also found that even smaller reductions (approximately 2 mmHg) in SBP resulted in a 7% decreased risk of coronary artery disease mortality and a 10% decreased risk of stroke mortality. Furthermore, Verdecchia et al. [45] reported that a 2 mmHg reduction in DBP leads to a 12% decreased risk of myocardial infarction, stroke, and cardiovascular mortality. Based on these previous findings, the results of the present study hold meaningful clinical significance.

Central hemodynamics represent the pressure in the ascending aorta and downstream (e.g., end organs). An elevation of the central blood pressure harms the vasculature and end organs, especially in the high blood pressure population [46]. Additionally, central blood pressure is recognized as a more powerful marker of end-organ damage and cardiovascular mortality compared to brachial blood pressure [47,48].

In the present study, 6 weeks of boxing training largely reduced cSBP (~8 mmHg) in individuals with elevated blood pressure or stage 1 hypertension. Similar to the findings from the present study, Beck et al. [49] reported a reduction in cSBP after 8 weeks of endurance (~11 mmHg) or resistance training (~10 mmHg) in young individuals with elevated blood pressure or stage 1 hypertension. This finding may be explained by an exercise-induced vasodilatory effect on resistance vessels in individuals with high blood pressure that reduced the magnitude of reflected pressure waves. Additionally, in the present study, no statistical reductions were observed in AIx or AIx@75 after 6 weeks of boxing training in individuals with elevated blood pressure or stage 1 hypertension. Previous studies have reported equivocal findings regarding the effects of exercise on AIx. Cheema et al. [12] reported a reduction in AIx after 16 weeks of boxing training in adults with abdominal obesity. Donley et al. [50] reported a reduction in cSBP and AIx@75 after 8 weeks of endurance training in adults with metabolic syndrome. Krustrup et al. [51] reported reductions in AIx after 12 and 24 weeks of soccer training in males with high blood pressure. Nualnim et al. [52] reported no significant reduction in AIx after 12 weeks of swimming training in elderly individuals with high blood pressure. Seals et al. [53] reported no changes in AIx after 12 weeks of walking training in older women with high blood pressure. Westhoff et al. [54] reported no changes in AIx and AIx@75 after upper-body endurance training in elderly individuals with high blood pressure. Lastly, Heffernan et al. [55] reported no changes in AIx following 12 weeks of resistance training in elderly individuals with high blood pressure. These incongruent results may be explained by the heterogeneity in exercise programs and sample characteristics (e.g., age, sex, morbidity) among studies.

Based on the findings of Vlachopoulos et al. [56], who reported that a 10 mmHg reduction in cSBP translates into an 8.8% decrease in the risk for future cardiovascular events, we propose that the findings of the present study are clinically relevant because a mean reduction of ~8 mmHg was observed after 6 weeks of boxing training in individuals with elevated blood pressure or stage 1 hypertension.

Finally, in the present study, no significant reduction in LVE_W_ (~37%) was observed after 6 weeks of boxing in individuals with elevated blood pressure or stage 1 hypertension. LVE_W_ is a relatively new biomarker that describes the additional energy required by the myocardium to overcome the augmented pressure generated by the reflected pressure wave. Thus, reductions in LVE_W_ may prevent the progression to pathological ventricular hypertrophy secondary to high blood pressure [57]. There are only a few studies that have explored the effects of exercise on LVE_W_. For example, Beck et al. [49] reported a reduction in LVEw after 8 weeks of endurance (~76%) or resistance training (~82%) in young individuals with elevated blood pressure or stage 1 hypertension. Based on these and the current study results, significant reductions in LVEw might be seen after more extended training periods.

### 4.2. Arterial Stiffness

Large-artery stiffness is an independent risk factor for cardiovascular mortality, and it is usually associated with high blood pressure [58]. In fact, it has been proposed that arterial stiffness is a mechanism behind the development or progression of high blood pressure [59,60]. PWVcf is the gold standard to assess arterial stiffness, and its reduction is associated with better cardiovascular outcomes [61,62].

In the present study, no changes in PWVcf were observed after 6 weeks of boxing training in individuals with elevated blood pressure and stage 1 hypertension. These findings agree with those of previous studies. Beck et al. [49] reported no changes in PWVcf after 8 weeks of endurance or resistance training in individuals with elevated blood pressure or stage 1 hypertension. Ferrier et al. [63] reported no PWVcf change following 8 weeks of cycling training in individuals with high blood pressure. Seals et al. [53] reported no effects on PWVcf after 12 weeks of endurance training in postmenopausal women with high blood. Stewart et al. [64] reported no significant changes in PWVcf after 24 weeks of endurance and resistance training in elderly individuals with high blood pressure in comparison to a control group. Lastly, Guimaraes et al. [65] reported a small reduction and no changes in PWVcf after 16 weeks of interval or continuous endurance training in individuals with high blood pressure.

In contrast, Madden et al. [66] reported ~3 m/s and ~1 m/s reductions in PWVcf after 12 and 24 weeks of endurance training in individuals with high blood pressure with other cardiovascular comorbidities. Interestingly, Collier et al. [67] reported that 4 weeks of resistance training increased PWVcf and 4 weeks of endurance training reduced PWVcf in individuals with high blood pressure. In a recent study, Bhuva et al. [68] reported a reduction in PWV across the length of the whole aorta measured by cardiovascular magnetic resonance after 24 weeks of endurance training in healthy sedentary individuals. Overall, exercise training programs shorter than 6 weeks are less likely to induce structural adaptations in elastic arteries in individuals with high blood pressure.

### 4.3. Vascular Adaptations

Exercise-induced vascular adaptations have been extensively reported in healthy and CVD individuals, including those suffering from high blood pressure [11,69]. These adaptations are vessel-specific (e.g., elastic, conduit, and resistance arteries) [11] and are activity-dependent (e.g., upper body versus lower body exercise) [70,71].

The present study demonstrated that 6 weeks of boxing training improves conduit artery endothelial function in individuals with elevated blood pressure or stage 1 hypertension. Specifically, brachial and popliteal mean FMD% increased by 3% and 2.6%, respectively. These findings were in agreement with the findings of Tinken et al. [72], who reported that brachial and popliteal FMD% increased by 1.7% and 1.6% in normotensive individuals after 6 weeks of endurance training. Similarly, Beck et al. [73] showed an increase in brachial FMD% after 8 weeks of endurance training (~3.7%) and after 8 weeks of resistance training (~2.1%) in individuals with elevated blood pressure or stage 1 hypertension. In the same study, Beck et al. described a blunted endothelial response to hyperemia in individuals with elevated blood pressure or stage 1 hypertension compared to normotensive matched controls before the exercise intervention. The findings of the present study were also in agreement with previous studies involving individuals with high blood pressure. For example, Westhoff et al. [54] showed a 2.3% increase in brachial FMD% after 12-week endurance training in elderly hypertensive individuals. Swift et al. [74] reported an increase in brachial FMD% ranging from 1% to 1.5% after 24 weeks of endurance training in postmenopausal women with hypertension. Nualnim et al. [52] reported a ~3.9% increase in brachial FMD% after 12 weeks of swimming training in adults > 50 years old with high blood pressure. Finally, Molmen-Hansen et al. [75] reported a 4.2% increase in brachial FMD% after 12 weeks of HIIT in individuals with high blood pressure. These FMD% improvements might be explained by an increase in ESS during exercise, which in turn increases NO and decreases inflammation [11,76,77,78]. Contrary to the present findings, no changes in brachial FMD% were reported by Westhoff et al. [79] in individuals with high blood pressure after 12 weeks of upper-body endurance exercise. Differences in endothelial responses in the present study and those of Westhoff et al. may be explained by a lower ESS stimulus produced during arm-cranking in comparison to whole-body exercise. For its part, Spence et al. [80] reported a significant improvement in brachial FMD% after 24 weeks of resistance training (~1.9%) but not after 24 weeks of endurance training in young healthy individuals, while no changes were observed in femoral FMD% with either training modality.

In addition, the present study showed equivocal effects on the structure of conduit arteries after 6 weeks of boxing training in individuals with elevated blood pressure or stage 1 hypertension. Specifically, there were significant increases in resting artery diameter in the common carotid artery (~0.3 mm) and in the popliteal artery (~0.2 mm) at the end of the intervention, but not in the brachial artery. Similar findings have been described in previous studies. Dinenno et al. [81] reported an increase in diameter in the femoral artery (~0.8 mm) but not in the brachial artery after ~14 weeks of walking or jogging training in normotensive sedentary individuals. Spence et al. [80] reported an increase in diameter in the brachial artery (~0.3 mm) with resistance training but no change with endurance training, an increase in diameter in the femoral artery diameter (~0.2 mm) with endurance training but not with resistance training, and no change in diameter in the carotid artery following either exercise training modality in young healthy individuals. In contrast, no exercise-induced structural adaptations have been described in previous studies that involved individuals with high blood pressure. For example, Nualnim et al. [52] reported no changes in brachial and carotid artery diameters after 12 weeks of swimming training in adults > 50 years old with high blood pressure. Beck et al. [73] reported no change in brachial artery diameter after 8 weeks of endurance or resistance training in individuals with elevated blood pressure or stage 1 hypertension. Finally, the findings of the present study were not entirely consistent with those of Tinken et al. [72], who reported no changes in brachial and popliteal diameters in normotensive individuals throughout 8 weeks of endurance training. However, Tinken et al. described a progressive increment in vasodilator capacity in both brachial and popliteal arteries starting from week 2 in the same study, which can be interpreted as an expression of artery remodeling. It has been suggested that adaptations of conduit arteries to exercise training begin with an increase in endothelial function during the first weeks, followed by artery remodeling (e.g., increase in diameter) to normalize resting ESS [11,72]. The results from the present study support this last theory, considering that resting ESS was not affected in the carotid and popliteal arteries after the boxing intervention despite the increase in diameters in both arteries.

Further, resistance vessel adaptations were also observed in the present study through venous occlusion plethysmography. The present boxing protocol increased baseline forearm blood flow (~27%), peak forearm blood flow (~22%), and peak calf blood flow (~26%). These changes reflected an augmented vascular ceiling capacity due to capillary or arteriolar proliferation and an improvement in endothelial function. These findings were in agreement with previous studies. Higashi et al. [82] reported an increase in peak forearm blood flow (~23%) in individuals with high blood pressure after 12 weeks of brisk walking. However, baseline forearm blood flow remained unchanged after the intervention. Beck et al. [32] reported an increase in baseline forearm blood flow (~22%), peak forearm flow (~31%), baseline calf blood flow (~33%), and peak calf blood flow (~44%) after 8 weeks of endurance or resistance training. Additionally, Beck et al. described that the endothelial function of resistance vessels was impaired in individuals with elevated blood pressure or stage 1 hypertension compared to normotensive controls. Based on previous studies, the findings from the present study could be explained by a reduction in sympathetic tone, metabolic changes (e.g., Angiotensin II and Endothelin-1 downregulation), and an increase in ESS [11].

### 4.4. Nitric Oxide Bioavailability

NO is a key molecule in the maintenance of vascular homeostasis and vascular health. NO is produced by eNOS from its substrate, L-arginine, in endothelial cells. One of the most important functions of NO is to act as a vasodilator by relaxing smooth muscle cells from the tunica media of an artery. Also, NO can inhibit platelet adhesion and aggregation, decrease ROS formation, and reduce inflammation. All these functions prevent endothelial dysfunction, which is the first step in the development of CVD [83].

As the NO half-life is relatively short, its direct quantification in blood is difficult. Estimations of NO production are best accomplished by quantification of the final NO metabolites nitrite/nitrate (NOx) [84]. The present study demonstrated that 6 weeks of boxing training in individuals with elevated blood pressure or stage 1 hypertension elevates NOx in plasma by nearly 27%. These results were in agreement with the findings of Beck et al. [73], who reported an increase in NOx after 8 weeks of cycling (~31%) and resistance training (~19%) in young individuals with elevated blood pressure or stage 1 hypertension. Similarly, Tomeleri et al. [84] reported an increase in NOx after 12 weeks of resistance training (~35%) in older women with high blood pressure, while Izadi et al. [40] reported an increment in NOx after 6 weeks of HIIT (~140%) in elderly individuals with high blood pressure. Additionally, Hasegawa et al. [85] reported an increase in NOx after 6 weeks of HIIT (~31%) and after 8 weeks of moderate-intensity cycling (~43%) in healthy young men. Moreover, Maeda et al. [86] reported an increase in NOx (~60%) following 12 weeks of cycling at 80% of the ventilatory threshold in healthy older women.

The potential mechanisms by which exercise might increase NO bioavailability in plasma are not yet fully understood. However, it is believed that vascular homeostasis is closely regulated by the mechanical interaction between blood flow and the endothelium through a process known as ESS [87]. During exercise, there is a rise in pulsatility and ESS inside arteries [11,88], which suggests that these hemodynamic changes may regulate the expression of genes involved in NO production through a mechanotransduction pathway [11,89,90,91,92].

### 4.5. Inflammation

Low-grade chronic inflammation has been described as a hallmark in the physiopathology of high blood pressure [93,94,95]. TNF-α, IL-6, and CRP are classic inflammatory biomarkers employed to explore the inflammatory state of an individual [96,97,98]. Several studies have shown a direct correlation between these biomarkers and high blood pressure [98,99,100,101]. TNF-α is a primary inflammatory cytokine released by white blood cells that stimulates visceral adipose tissue to secrete IL-6, a secondary inflammatory cytokine, which in turn increases the production and excretion of CRP from the hepatocyte [100,102,103]. Additionally, CRP can be secreted by other cell types, including endothelial cells [104], where it has been shown to have deleterious autocrine and paracrine effects on them (e.g., overexpression of adhesion molecules) [105,106,107]. In the present study, CRP decreases after 6 weeks of boxing training, but neither TNF-α nor IL-6 does. These findings can be explained by the fact that a short period of exercise training (e.g., 6 weeks) by itself does not significantly affect body fat mass and specifically the amount of adipose tissue; consequently, there may be no significant modification in the adipocyte signaling pathway to alter TNF-α and IL-6 production. Conversely, CRP reductions could be the result of an exercise-induced signal (e.g., mechanotransduction or myokines) that directly enhances endothelial function and inhibits CRP secretion into the bloodstream. A reduction in CRP may indicate a direct downregulation of vascular inflammation, which in turn helps prevent high blood pressure.

The results of the present study were in agreement with those of Lamina et al. [108], who reported a CRP reduction after 8 weeks of cycling training at moderate intensity in individuals with high blood pressure. Interestingly, baseline CRP levels differed between studies. While Lamina et al.’s study reported a baseline mean CRP value below 1 mg/L, which is considered to be in the non-inflammatory range, the present study showed a baseline mean CRP value above 3 mg/L, which is categorized as high risk for the development of cardiovascular events [107,109]. These results confirm that exercise can be employed as an anti-inflammatory strategy for individuals with high blood pressure.

### 4.6. Oxidative Stress

Oxidative stress is another feature associated with the pathophysiology of high blood pressure [4,95,110]. In the present study, no changes were observed in oxidative stress status as observed by the measurements of 8-isoprostane, SOD, and TAC in the plasma of individuals with elevated blood pressure or stage 1 hypertension after 6 weeks of boxing training. These findings were incongruent with previous studies. Feairheller et al. [111] reported an increase in plasma TAC (~9%) and urinary 8-isoprostane (~31%) after 24 weeks of endurance training at 70% VO_2_max in individuals with high blood pressure. Beck et al. [32] reported a reduction in plasma 8-isoprostane (~43%) and an increase in plasma TAC (~43%) after 8 weeks of resistance training and a reduction in plasma 8-isoprostane (~40%) and an increase in plasma TAC (~42%) after 8 weeks of endurance training in individuals with elevated blood pressure and stage 1 hypertension. Lastly, Dantas et al. [112] reported an increase in plasma TAC (~12%) after 12 weeks of resistance training in older women with high blood pressure. Differences in the oxidative stress responses between the present study and previous studies may be explained by the length of each exercise program. The present study involved only 6 weeks of training, while the other studies lasted from 8 to 24 weeks.

Additional analyses were performed to evaluate whether sex or adiposity may have confounded the intervention outcomes. The correlations between sex, baseline BMI, ΔBMI, and all primary variables were consistently weak and non-significant (Supplementary Table S1). These results suggest that the improvements observed in blood pressure, endothelial function, and biochemical markers were not attributable to differences in adiposity or sex. Although exercise training can promote weight loss that may contribute to blood pressure reductions, the ΔBMI values in the present study were modest and did not correlate significantly with changes in SBP, DBP, or vascular outcomes. Taken together, the data reinforce that the beneficial effects of the intervention are more likely due to the physiological stimulus of training rather than changes in body weight or demographic differences.

The major strengths of this study include its randomized controlled design, the use of objective and gold-standard vascular measures (FMD, PWA, PWV, and VOP), and highly adherent training in the intervention group. However, the present study was not without limitations. First, only young adults (<35 years old) participated in this study, which may limit the generalization of the present results to only young individuals with elevated blood pressure or stage 1 hypertension. Second, a dietary record was not employed to ensure that a low-nitrate diet was followed prior to blood collection, which could have affected the results of some biomarkers. Third, structural adaptations in muscular arteries may be better represented by the conduit dilator capacity rather than the artery diameter, which was not measured in the present study. Lastly, a key limitation of this study is the disparity in adherence between the Boxing (98.1%) and Control (27.3%) groups. The significantly lower adherence in the Control group, potentially due to the inherent lack of engagement in a non-exercise, stretching/balance protocol, may introduce bias. Furthermore, the difference in adherence levels means there was likely an unequal effect of investigator contact and supervision between groups, which could have partially accounted for the observed changes in the intervention group. We acknowledge this as a limitation; however, the magnitude of the cardiovascular and vascular improvements observed strongly suggests that the physiological effects of the high-intensity boxing training were the primary drivers of the positive outcomes. It is important to note that the methodology used for cardiovascular risk stratification in this study precedes the release of the updated 2025 AHA/ACC BP guidelines, which now recommend utilizing the PREVENT risk calculator instead of the Pooled Cohort Equations for risk assessment. Future boxing training studies in individuals with elevated blood pressure or stage 1 hypertension should include an elderly population or be part of a community-based program to determine if the results of the present study can be generalized.

## 5. Conclusions

In conclusion, the present study demonstrated that a 6-week, 3 days per week boxing training program successfully reduced SBP, DBP, and cSBP while improving vascular health in individuals with elevated blood pressure or stage 1 hypertension. These effects may be explained, in part, by a reversal of some pathological pathways involved with high blood pressure, such as endothelial dysfunction, NO bioavailability, peripheral vascular resistance, and inflammation. Further, boxing training could be recommended as an exercise alternative in the management of the early stages of high blood pressure.

## Figures and Tables

**Table 1:** Demographic characteristics of the participants.

	Boxing Training Group			Control Group			
	Male	Female	Total	Male	Female	Total	*p*
	*n* = 8	*n* = 4	N = 12	*n* = 7	*n* = 4	N = 11	
**Age (y)**	27.6 (6.7)	23.5 (0.6)	26.3 (5.7)	26.0 (4.9)	22.0 (0.8)	24.6 (4.3)	0.235
**Height (m)**	1.7 (0.1)	1.5 (0.1)	1.7 (0.1)	1.7 (0.1)	1.6 (0.1)	1.7 (0.1)	0.698
**Weight (kg)**	97.7 (24.9)	72.4 (17.9)	89.3 (25.2)	100.6 (17.9)	88.8 (11.7)	96.3 (16.4)	0.442
**Hct (%)**	49.3 (3.4)	46.0 (3.3)	48.0 (3.7)	49.0 (2.9)	46.0 (4.2)	47.9 (3.5)	0.953
**SBP (mmHg)**	130.0 (7.2)	113.3 (4.6)	124.4 (10.3)	124.7 (13.3)	110.0 (11.8)	119.4 (14.3)	0.339
**DBP (mmHg)**	84.9 (6.1)	83.8 (2.5)	84.5 (5.1)	83.9 (4.7)	84.0 (4.1)	83.9 (4.2)	0.766

Data expressed as mean (SD). Hct: Hematocrit; SBP: systolic blood pressure; DBP: diastolic blood pressure.

**Table 2:** Pulse wave analysis and arterial stiffness changes following 6 weeks of intervention.

	Boxing Training			Control			Interaction	Group	Time
	Before	After	Δ	Before	After	Δ	*p*	*p*	*p*
SBP (mmHg)	124.4 (10.3)	108.4 (7.7)	−16.0 (8.7)	119.4 (14.3)	122.2 (13.5)	2.8 (7.0)	<0.001	0.351	0.001
DBP (mmHg)	84.5 (5.1)	74.2 (5.0)	−10.3 (4.6)	83.9 (4.3)	84.2 (6.0)	0.3 (3.3)	<0.001	0.026	<0.001
cSBP (mmHg)	114.5 (10.7)	106.5 (6.3)	−7.9 (9.0)	113.9 (11.0)	112.5 (10.0)	−1.4 (5.6)	0.050	0.476	0.008
cDBP (mmHg)	80.8 (9.0)	75.7 (8.0)	−5.1 (7.0)	79.0 (9.7)	77.2 (11.2)	−1.8 (6.0)	0.231	0.972	0.020
cPP (mmHg)	33.6 (4.1)	31.0 (3.6)	−2.8 (3.8)	34.8 (3.3)	33.8 (7.1)	−1.0 (7.3)	0.479	0.207	0.129
AIx (%)	15.0 (10.3)	5.6 (17.4)	−9.3 (12.7)	16.8 (12.1)	13.5 (7.0)	−3.4 (15.0)	0.314	0.271	0.040
AIx@75 (%)	12.3 (11.6)	3.3 (17.3)	−9.0 (11.8)	16.9 (12.7)	10.4 (7.4)	−6.5 (14.9)	0.658	0.213	0.011
LVE_W_ (dynes/s/cm^2^)	1217.5 (509.1)	764.9 (606.1)	−452.7 (351.5)	1422.0 (607.5)	1132.6 (415.0)	−289.4 (747.6)	0.504	0.150	0.006
PWV *cf* (m/s)	6.7 (1.3)	6.5 (1.2)	−0.2 (0.4)	6.4 (1.5)	6.5 (1.2)	0.2 (0.7)	0.096	0.797	0.779

Data expressed as mean (SD). SBP: systolic blood pressure; DBP: diastolic blood pressure; cSBP: central systolic blood pressure; cDBP: central diastolic blood pressure; cPP: pulse pressure; AIx: augmentation index; AIx@75: augmentation index at 75 beats per minute; LVEW: left ventricular energy wasted; PWVcf: pulse wave velocity carotid–femoral.

**Table 3:** Vascular changes following 6 weeks of intervention.

	Boxing Training			Control			Interaction	Group	Time
	Before	After	Δ	Before	After	Δ	*p*	*p*	*p*
Brachial Diameter (mm)	3.7 (0.6)	3.8 (0.7)	0.1 (0.2)	3.9 (0.6)	3.8 (0.7)	−0.1 (0.2)	0.095	0.881	0.895
Resting Brachial ESS (dynes/cm^2^)	31.7 (6.2)	30.0 (6.9)	−1.7 (5.4)	31.6 (6.4)	28.1 (5.1)	−3.5 (4.5)	0.398	0.674	0.022
Brachial FMD (mm)	0.30 (0.14)	0.42 (0.17)	0.12 (0.07)	0.30 (0.1)	0.25 (0.11)	−0.05 (0.08)	<0.001	0.338	<0.001
Brachial FMD (%)	8.5 (4.8)	11.5 (5.4)	3.0 (2.3)	8.2 (3.4)	7.0 (3.6)	−1.2 (1.9)	<0.001	0.191	0.061
Popliteal Diameter (mm)	5.5 (1.1)	5.8 (1.1)	0.2 (0.2)	6.6 (1.0)	6.5 (0.9)	−0.1 (0.3)	0.015	0.108	0.411
Resting Popliteal ESS (dynes/cm^2^)	19.3 (3.0)	20.5 (3.0)	1.1 (2.6)	22.2 (6.6)	21.6 (4.6)	−0.6 (4.5)	0.354	0.336	0.760
Popliteal FMD (Δ mm)	0.46 (0.16)	0.62 (0.18)	0.16 (0.09)	0.53 (0.32)	0.45 (0.28)	−0.08 (0.12)	<0.001	0.349	<0.001
Popliteal FMD (%)	8.4 (2.6)	11.0 (3.3)	2.6 (1.6)	8.2 (5.0)	7.0 (4.2)	−1.2 (2.0)	0.001	0.265	0.12
Carotid Diameter (mm)	6.7 (0.6)	7.0 (0.6)	0.3 (0.2)	7.2 (0.5)	7.0 (0.6)	−0.2 (0.3)	0.001	0.348	0.301
Resting Carotid ESS (dynes/cm^2^)	32.4 (5.3)	34.4 (5.9)	2.0 (5.1)	33.3 (7.5)	32.6 (6.6)	−0.7 (5.5)	0.228	0.836	0.572
VOP Forearm Basal (ml/min/100 mL tissue)	2.6 (0.8)	3.3 (1.0)	0.7 (0.7)	3.3 (0.9)	3.2 (1.1)	−0.1 (0.4)	0.003	0.469	0.019
VOP Forearm Peak (ml/min/100 mL tissue)	17.1 (4.5)	20.9 (5.7)	3.8 (2.6)	14.9 (3.9)	14.8 (3.9)	−0.1 (1.3)	<0.001	0.037	<0.001
VOP Calf Basal (ml/min/100 mL tissue)	2.7 (0.5)	3.0 (0.5)	0.3 (0.6)	3.2 (0.6)	3.2 (0.6)	0.0 (0.5)	0.134	0.143	0.126
VOP Calf Peak (ml/min/100 mL tissue)	17.6 (4.3)	22.1 (6.2)	4.5 (4.6)	17.4 (6.9)	17.4 (6.2)	0(1.2)	0.005	0.305	0.005

Data expressed as mean (SD). ESS: endothelial shear stress; FMD: flow mediated dilation; VOP: venous occlusion plethysmography.

**Table 4:** Blood biomarker changes following 6 weeks of intervention.

	Boxing Training			Control			Interaction	Group	Time
	Before	After	Δ	Before	After	Δ	*p*	*p*	*p*
NOx (μmol/L)	53.9 (19.6)	68.4 (23.3)	14.5 (17.4)	60.7 (19.9)	52.7 (26.5)	−8.0 (27.0)	0.005	0.627	0.370
CRP (mg/L)	9.1 (7.3)	5.9 (5.1)	−3.2 (3.5)	6.3 (4.4)	6.6 (4.6)	0.3 (2.3)	0.010	0.643	0.028
IL-6 (pg/mL)	19.1 (18.3)	19.0 (15.3)	−0.1 (5.7)	15.3 (13.8)	14.7 (14.9)	−0.6 (7.0)	0.851	0.546	0.810
TNFα (pg/mL)	126.9 (185.7)	134.1 (183.6)	7.2 (14.5)	91.4 (138.5)	91.1 (139.7)	−0.3 (9.0)	0.203	0.605	0.244
8-isoprostane (pg/mL)	465.1 (108.0)	487.3 (139.7)	22.2 (90.1)	440.2 (142.2)	535.7 (159.2)	95.5 (245.8)	0.364	0.792	0.152
SOD (mU/mL)	44.5 (6.2)	44.7 (6.4)	0.3 (2.2)	43.7 (6.5)	43.5 (6.9)	−0.3 (4.7)	0.731	0.710	1.000
TAC (mM/mL)	5.6 (2.5)	6.8 (3.5)	1.2 (2.4)	5.2 (2.7)	4.8 (2.0)	−0.3 (2.6)	0.151	0.242	0.408

Data expressed as mean (SD). NOx: nitric oxide; CRP: C-reactive protein; IL: interleukin; SOD: superoxide dismutase; TAC: total antioxidant capacity.

## Data Availability

The datasets employed in this study can be obtained from the corresponding author upon reasonable request. However, certain data cannot be made publicly accessible due to privacy considerations.

## Supplementary Materials

The following supporting information can be downloaded at https://www.mdpi.com/article/10.3390/sports14010005/s1, Table S1: Correlations between sex, baseline BMI, ΔBMI, and primary vascular outcomes.

## Abbreviations

The following abbreviations are used in this manuscript:

ACC/AHA	American College of Cardiology/American Heart Association
AIx	Augmentation index
AIx@75	Augmentation index normalized to a heart rate of 75 bpm
ANOVA	Analysis of variance
CBF	Calf blood flow
cSBP	Central systolic blood pressure
cDBP	Central diastolic blood pressure
cPP	Central pulse pressure
cAIx	Central augmentation index
CVD	Cardiovascular diseases
D	Diameter
DBP	Diastolic blood pressure
ESS	Endothelial shear stress
FBF	Forearm blood flow
FMD	Flow-mediated dilation
LVEW	Left ventricular energy
NO	Nitric oxide
NOx	Nitric oxide metabolites nitrite/nitrate
PP	Pulse pressure
PWA	Pulse wave analysis
PWVcf	Pulse wave velocity carotid–femoral
RPE	Rate of perceived exertion
SBP	Systolic blood pressure
SD	Standard deviation
VOP	Venous occlusion plethysmography
