# Supplementary material for: Six Weeks of Boxing Training Lowers Blood Pressure and Improves Vascular Function in Young Men and Women with Elevated Blood Pressure or Stage 1 Hypertension
Source: Sports (Basel). 2026 Jan 1;14(1):5. doi: 10.3390/sports14010005 (PMC12846006; doi:10.3390/sports14010005)
Supplement: Supplementary file 1 [file sports-14-00005-s001.zip › sports-3976344-supplementary.pdf]

Table S1: Correlations between sex, baseline BMI, ΔBMI, and primary vascular outcomes.

Predictor	Outcome	*r*	*p*
Sex	ΔSBP	0.302	0.161
	ΔDBP	0.252	0.245
	ΔBFMD%	0.011	0.961
	ΔPFMD%	0.184	0.496
	ΔNOx	0.213	0.342
	ΔCRP	-0.002	0.921
Baseline BMI	ΔSBP	0.004	0.986
	ΔDBP	0.187	0.393
	ΔBFMD%	-0.187	0.392
	ΔPFMD%	-0.242	0.366
	ΔNOx	-0.106	0.640
	ΔCRP	0.127	0.572
ΔBMI	ΔSBP	0.319	0.138
	ΔDBP	0.324	0.132
	ΔBFMD%	-0.362	0.089
	ΔPFMD%	-0.466	0.069
	ΔNOx	-0.182	0.416
	ΔCRP	0.311	0.159

*BMI: body mass index; ΔBMI: delta body mass index; ΔSBP: delta systolic blood pressure; ΔDBP: delta systolic blood pressure; ΔBFMD%: delta brachial flow-mediated dilation; ΔPFMD%: popliteal flow-mediated dilation; ΔNOx: delta nitric oxide; ΔCRP: delta C-reactive protein.
